# Understanding care coordination for Veterans with complex care needs: protocol of a multiple-methods study to build evidence for an effectiveness and implementation study

**DOI:** 10.3389/frhs.2023.1211577

**Published:** 2023-08-15

**Authors:** Denise M. Hynes, Diana J. Govier, Meike Niederhausen, Anaïs Tuepker, Avery Z. Laliberte, Holly McCready, Alex Hickok, Mazhgan Rowneki, Dylan Waller, Kristina M. Cordasco, Sara J. Singer, Kathryn M. McDonald, Christopher G. Slatore, Kathleen C. Thomas, Matthew Maciejewski, Catherine Battaglia, Lisa Perla

**Affiliations:** ^1^ Center to Improve Veteran Involvement in Care (CIVIC), VA Portland Health Care System, Portland, OR, United States; ^2^ School of Nursing, Oregon Health & Science University, Portland, OR, United States; ^3^ College of Public Health and Human Sciences, Oregon State University, Corvallis, OR, United States; ^4^ School of Public Health, Oregon Health & Science University & Portland State University, Portland, OR, United States; ^5^ Department of Family Medicine, Oregon Health & Science University, Portland, OR, United States; ^6^ Center for the Study of Healthcare Innovation, Implementation & Policy (CSHIIP), VA Greater Los Angeles Healthcare System, Los Angeles, CA, United States; ^7^ Department of Medicine, David Geffen School of Medicine at the University of California, Los Angeles, CA, United States; ^8^ Department of Medicine, Stanford University School of Medicine, Stanford, CA, United States; ^9^ Center for Diagnostic Excellence, Armstrong Institute for Patient Safety and Quality, Johns Hopkins School of Nursing, Baltimore, MD, United States; ^10^ Division of General Internal Medicine, Department of Medicine, Johns Hopkins School of Medicine, Baltimore, MD, United States; ^11^ Division of Pulmonary & Critical Care Medicine, Department of Medicine, Oregon Health & Science University, Portland, OR, United States; ^12^ Section of Pulmonary & Critical Care Medicine, VA Portland Health Care System, Portland, OR, United States; ^13^ Division of Pharmaceutical Outcomes and Policy, University of North Carolina at Chapel Hill, Chapel Hill, NC, United States; ^14^ Center of Innovation to Accelerate Discovery and Practice Transformation (ADAPT), Durham Veterans Affairs Health Care System, Durham, NC, United States; ^15^ Department of Population Health Sciences & Division of General Internal Medicine, Department of Medicine, Duke University, Durham, NC, United States; ^16^ Department of Veterans Affairs, Eastern Colorado Health Care System, Denver, CO, United States; ^17^ Department of Health Systems, Management & Policy, University of Colorado Anschutz Medical Campus, Aurora, CO, United States; ^18^ Rehabilitation Services, Veterans Affairs Central Office, Washington, DC, United States

**Keywords:** Veterans, care coordination, study protocol, access to care, care integration

## Abstract

**Background:**

For patients with complex health and social needs, care coordination is crucial for improving their access to care, clinical outcomes, care experiences, and controlling their healthcare costs. However, evidence is inconsistent regarding the core elements of care coordination interventions, and lack of standardized processes for assessing patients’ needs has made it challenging for providers to optimize care coordination based on patient needs and preferences. Further, ensuring providers have reliable and timely means of communicating about care plans, patients’ full spectrum of needs, and transitions in care is important for overcoming potential care fragmentation. In the Veterans Health Administration (VA), several initiatives are underway to implement care coordination processes and services. In this paper, we describe our study underway in the VA aimed at building evidence for designing and implementing care coordination practices that enhance care integration and improve health and care outcomes for Veterans with complex care needs.

**Methods:**

In a prospective observational multiple methods study, for Aim 1 we will use existing data to identify Veterans with complex care needs who have and have not received care coordination services. We will examine the relationship between receipt of care coordination services and their health outcomes. In Aim 2, we will adapt the Patient Perceptions of Integrated Veteran Care questionnaire to survey a sample of Veterans about their experiences regarding coordination, integration, and the extent to which their care needs are being met. For Aim 3, we will interview providers and care teams about their perceptions of the innovation attributes of current care coordination needs assessment tools and processes, including their improvement over other approaches (relative advantage), fit with current practices (compatibility and innovation fit), complexity, and ability to visualize how the steps proceed to impact the right care at the right time (observability). The provider interviews will inform design and deployment of a widescale provider survey.

**Discussion:**

Taken together, our study will inform development of an enhanced care coordination intervention that seeks to improve care and outcomes for Veterans with complex care needs.

## Introduction

Care coordination has proved essential for improving patients’ access to care (1), clinical outcomes (2–4), and experiences of care (3, 5–7), as well as increasing provider satisfaction (8) and decreasing or controlling healthcare costs (7, 8), including among Veterans receiving care from the US Department of Veterans Affairs (VA) health care facilities (5, 7, 9). Although evidence to date is inconsistent regarding the design and effectiveness of tools to assess care coordination needs (10–12), care coordination appears to be most successful for patients with conditions that increase their risk for hospitalization, mortality and higher costs (3, 9, 13, 14). Such high-risk, high need populations are not limited to those with a specific set of comorbidities and may include people with health-related social needs (15, 16). Care coordination for people with complex care needs is multidimensional and spans a continuum of care (17–19). It encompasses reliable and valid patient assessment; effective communication between patient, caregiver, and providers; and linkages between the family, community, and healthcare system (17, 20). Integration across domains of care is needed, rather than separate care coordination layers (21).

In the VA, evidence on care coordination for patients with complex care needs has been emerging. In the randomized trial of enhanced care coordination services within the VA Patient Aligned Care Team (PACT) Intensive Management (PIM) model, adding care coordination to the established and effective PACT model (1, 7, 22, 23) increased patients’ primary care and social work services, and outpatient healthcare costs. The intervention was also associated with decreased inpatient costs, resulting in similar total costs of care (7) and modest improvements in patient experiences of care (5). Bauer and colleagues demonstrated in a randomized stepped-wedge implementation trial that incorporating a facilitator into the mental health team led to decreased mental health hospitalizations for patients with serious mental illness, and for those with more complex conditions, improved mental health component scores as well (9). Attention to the unique and complex needs of women Veterans also highlights the potential for care coordination to improve pregnancy and birth outcomes (24–26), and Medicare patients have benefited from care coordination after hospitalization through reduced mortality and healthcare costs (27). Receiving care in an integrated care setting like the VA when there are multiple providers might appear to be fragmented. However, integrated care with multiple providers may minimize hospitalizations for ambulatory care sensitive conditions, and enhance access to more timely or specialized care (28).

Despite this evidence, the extent to which care coordination is contributing to outcomes is not fully known. The core elements of care coordination interventions for patients with complex care needs have varied considerably across interventions and patient populations (20, 21). Core elements of care coordination interventions as described in a 2018 VA State of the Art Conference on Care include the contextual factors, locus (setting, level, and purpose), and design features (mechanisms and types of coordination such as structural, functional, normative, interpersonal, and clinical) and are essential in the design and evaluation of care coordination interventions (29, 30). In practice, lack of standardized needs assessment tools and processes has made it challenging for healthcare providers to determine the optimal amount and type of care coordination services based on patient needs and preferences (31), and thus to measure the dose-response relationship. Ideally, care coordination ensures that accountable structures and processes are in place for communication and integration of a comprehensive care plan across providers and settings that is aligned with patient and family needs, preferences, and goals (32). Further, ensuring that engaged care providers have reliable and timely means of communication about care plans is essential to inform provider actions and to overcome the potential for care fragmentation. A key challenge in delivering integrated patient care is to optimally balance coordination and patient centeredness.

The purpose of this paper is to outline what is known and not known about care coordination and its impacts among Veterans and describe our study underway in the VA. We will conduct a prospective observational multiple methods study, which will gauge the relationship between care coordination services and subsequent healthcare use among Veterans with complex care needs, assess Veterans’ perspectives about care integration in the services they have received, and assess provider perspectives about care integration and care coordination tools and processes. We describe potential limitations to our approach as well. Finally, we highlight how our approach may inform evidence building in other health care systems that can inform implementation of an improved care model that incorporates these perspectives, addresses the identified needs, and focuses care on aspects with the greatest potential to improve patient outcomes.

### Theoretical framework and logic model

In our prior work, we explored the linkages between care coordination theory and practice and considered three specific domains—the context surrounding an intervention, an intervention’s locus (setting, level, and purpose), and elements of its design (mechanisms and types of interventions) (29, 30). The linkages from theory to practice underpin measurement development and testing, which is important for evaluating care coordination processes and intermediate outcomes, and patient experiences of care coordination interventions.

**Figure 1:**
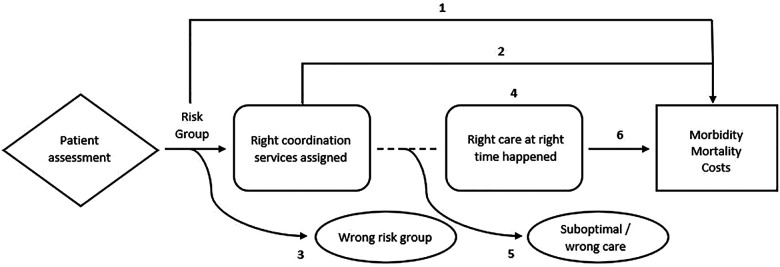
Logic model: operationalizing care coordination process & outcomes.

To operationalize and develop measures to examine how care coordination relates to patient outcomes, we viewed care coordination as a process with a sequence of steps and corresponding outcomes to be measured. Figure 1 depicts components in a graphical chain of logic with a diamond box for care coordination tools and processes for assessing patient needs, arrows for the process flow, dotted lines for associations, rounded boxes for intermediate outcomes, rectangles for health states, and ovals for harms. In this case, the process starts at the point of assessing a person's needs and risks and posits the cause-and-effect relationships from that point onward that may result in favorable or unfavorable systems-level and patient-level outcomes, and informs key questions, such as:

1. At a population level (and without intervention), what are the predicted outcomes for each risk group as determined by a care coordination needs assessment?
2. What is the effect of a needs-assessment-determined risk group intervention (assigned care coordination resources) on outcomes (e.g., morbidity, mortality, health care use and costs).
3. In the absence of intervention, what proportion of patients are categorized into the “wrong” risk group (and proceed to have outcomes at different levels/rates than expected)?
4. What are the associations between care coordination services and receiving the “right care at the right time”?
5. Even with care coordination services, what proportion of patients do not receive any care or optimal care (“suboptimal/wrong care”)?
6. What is the effect of “right care at the right time” on outcomes (e.g., morbidity, mortality, health care use and costs)?

### Study aims

With this guiding logic model, our multiple methods study comprises three aims intended to begin to address some but not all aspects. For Aim 1, we will focus on components 1–3 in the logic model by characterizing and comparing relationships between care coordination processes and health and care outcomes among Veterans with complex care needs. We will begin to address components 4 and 5 by surveying Veterans about their experiences regarding care coordination and the extent to which their needs were met (Aim 2). We will also interview and survey providers (Aim 3) about their perceptions of the attributes of care coordination needs assessment tools and processes that VA sites have implemented, including their improvement over other approaches (relative advantage), their fit with current practices at their facility (compatibility and innovation-fit), complexity, and the ability to visualize how the steps proceed to impact the right care at the right time (observability). To assess the extent to which the right care happens at the right time to potentially impact outcomes (component 6), we will combine information gathered from all three aims to describe scenarios that will exemplify the need for a future implementation study. Taken together our approach will inform development of enhanced care coordination processes with a goal of improving care integration and patient outcomes.

## Methods and analysis

As a multiple methods study, we have three aims, each with specific research questions, hypotheses, and design aspects. The design and methods for each study aim follows.

### Aim 1: relationships between process and outcomes

#### Research questions and hypotheses

In aim 1 our main research question is “Are care coordination services associated with differences in health and care outcomes, including emergency department (ED) and urgent care (UC) visits, hospitalizations, mortality, and healthcare costs among Veterans with complex care needs?” We hypothesize that receipt of care coordination services will be associated with fewer ED/UC visits and hospitalizations, decreased mortality, and reduced or neutral healthcare costs among these Veterans.

#### Objective and study sample

We will characterize and compare the relationship between receiving care coordination services and subsequent healthcare use, health outcomes, and healthcare costs among Veterans with high-risk, high-need conditions in a prospective observational study. With existing data available in the VA's electronic health record (EHR), VA-purchased care claims, and Medicare claims data from the Centers for Medicare and Medicaid Services (CMS), we will identify and match Veterans with complex care needs with and without care coordination services based on clinical and sociodemographic characteristics and prior healthcare use. The VA lacks a standardized care needs assessment in the EHR, but does include a means to identify Veterans with high risk for hospitalization and/or mortality, based on annual care assessment of needs (CAN) scores (33). Those with a CAN score of 85 or greater, indicating their predicted probability for hospitalization and/or mortality at one-year is at or above the 85th percentile, will be included. The CAN score is generated from VA EHR data and includes information on sociodemographic characteristics, chronic illness measures (including the Deyo-Charlson comorbidity index), VA healthcare use, vital sign parameters, laboratory test values, specific medication use, and 18 drug interactions. The CAN score has been well validated for predicting hospitalization and mortality and is in wide use in the VA. Use of the CAN score allows us to identify those most likely to benefit from care coordination and explore the relationships with processes and outcomes. Exclusion criteria include CAN score less than 85 and missing data for key variables required for matching. We will follow Veterans with complex care needs, with and without care coordination services over time to examine and compare their healthcare use, costs, and health outcomes.

To assess use of care coordination services, we will rely on information from VA documentation of service provision. In the VA, care coordination is documented as specific services in accordance with CMS coding and using Current Procedural Terminology (CPT) codes, including transitional care management, chronic care management and complex chronic care management codes and with detailed requirements for who can deliver the services and how frequently the services can be reported for CMS. VA care is not subject to the CMS frequency limits, however. Table 1 provides information on the CPT code categories and types of care coordination that will be used in our study.

**Table 1:** CPT codes for care coordination services, description of services and allowed billing frequency based on Medicare requirements.

Code type	CPT code	Description	Providers who can deliver service	Allowed frequency of billing
TCM Codes	Code 99496	Transitional care management services with moderate medical decision complexity	Physician or non- physician practitioner such as PA, NP, CNS, and CNM	At post-hospital discharge; visit within 7 days of discharge
	Code 99495	Transitional care management services with high medical decision complexity		At post-hospital discharge; visit within 14 days of discharge
CCM	Code 99490	Chronic care management for a patient with multiple chronic conditions	Physician or non- physician practitioner such as PA, NP, CNS, and CNM, and their clinical staff such as RN, SW, and LPN	Once per calendar month; at least 20 min of time spent with patient per month
CCCM Codes	Code 99487	Complex chronic care management for a patient with multiple chronic conditions		Once per calendar month; 60 min of time spent with patient per month
CCM Codes	Code 99489	Each additional 30 min of time spent per month with patient on complex chronic care management services	Physician or non- physician practitioner such as PA, NP, CNS, and CNM	Once per calendar month
	Code G0506	Add on to initial CCM visit At time of in-person visit when providing care plan. Can be billed only once		At time of in- person visit when providing care Plan; can be billed only once
	Code T1016	Case management services		15-minute intervals; only used in VA, no longer used by CMS since 2013

Medicare Learning Network (34); Isetts et al. (35); Rivera et al. (36).

TCM, transition care management; CCM, chronic care management; CCCM, complex chronic care management; PA, physician assistant; NP, nurse practitioner; CNS, certified nurse specialist; CNM, certified nurse midwife; RN, registered nurse; SW, social worker; LPN, licensed practical nurse; VA, Veterans Affairs.

In planning this study, we queried the VA Corporate data warehouse to gauge the volume of care coordination workload in selected sites involved in the early phase of VA Care Coordination and Integrated Case Management initiative, which aimed to begin standardizing care coordination assessment across VA facilities (37). For Aim 1 we will include all VA facilities that offer care coordination services.

#### Outcome measures

Pilot data from FY2018 from one VA medical center were used to estimate a conservative sample size of cases (those with a needs assessment documented) and comparator patients for at least 12 VA facilities. We estimated a minimum of 88 cases per site; and with a 1:1 match for comparators, a total sample of 2,112. With growth in the number of VA sites expanding and documenting care coordination services, we expect this number to be easily surpassed during the observation period (FY2019–FY2021).

#### Emergency Department (ED) visits

In the pilot data, there were an average of 15.5 ED visits per year in Veterans with care coordination. We estimated Veterans without care coordination to have 26 ED visits per year. With an estimated sample size and ED visit rates, we calculated 98.6% power to detect a difference of 20% in ED visits per year between those with and without care coordination, which is in line with prior studies (38).

#### Healthcare costs

We will estimate annual total healthcare costs (VA and Medicare), including outpatient, ED, UC, inpatient, and medication costs. For power calculation estimates, we rely on Yoon et al. (7), which indicates unadjusted VA costs for complex patients at $31,956 (95% CI $29,840, $34,443) in 2015 dollars. We estimated that we will have a minimum of 85.1% power to detect an effect size of 0.13–0.20 between Veterans with and without care coordination. We have considered the anticipated larger variance in costs expected as reported in prior research for cost estimations that include both VA and Medicare costs (1, 39, 40).

#### Mortality

We considered experience of Wang et al. (33), and our own preliminary analysis of primary care patients with risk scores indicating high-risk, in which yearly mortality rates ranged from 6.2% to 7.9%. Using data from VA and CMS mortality sources (41), conservatively, using a 5% mortality rate among Veterans with care coordination, we have 81% power to detect a significant odds ratio (OR) of 1.6, with power increasing to 88% if the mortality rate among Veterans without care coordination is 8%.

#### Analysis

We will describe and compare Veterans with complex care needs, with and without care coordination services along each outcome measure using descriptive statistics and visualizations including counts and proportions for categorical variables, and means, standard deviations, ranges, and quartiles for continuous variables, as well as 95% confidence intervals (CI) for all proportions and means. Unadjusted comparisons between the two groups will be tested using *t*-tests or Chi-squared tests. We will test the hypothesis that outcomes improved among Veterans with care coordination compared with Veterans without care coordination using regression models and an appropriate matching (e.g., propensity score matching) approach (42). As an observational study in which we are interested in assessing treatment effects, matching will allow us to compare outcomes for Veterans with and without care coordination services. Regression models planned include generalized linear mixed models (GLMM), logistic GLMM, and generalized estimated equations (GEE). Cost outcomes will be modeled with GEE to provide flexibility in choice of distribution and link function (43).

### Aim 2: Veterans' perspectives

#### Research question and hypotheses

For Aim 2, the main research question is “Are care coordination services associated with differences in perceived care integration and patient centeredness of care among Veterans with complex care needs? We hypothesize that Veterans with high-risk, high-need conditions with complex care needs who receive care coordination services will report greater levels of care integration and patient-centeredness than those who do not receive these services.

#### Objective and study sample

We will assess Veterans’ perspectives about their experiences with care coordination, integration with other healthcare services, and perceived health impacts. We will sample and recruit participants from among Veterans identified in Aim 1 from FY2021 and based at VA sites with active care coordination programs (29 sites). Initially approved for a sample of 2,500 Veterans, we will invite participants from the selected sites and using a proportionate random sample of those with and without care coordination services.

#### Measures

Measures will be drawn from an adaptation of Singer's Framework for Measuring Integrated Patient Care (32) and the Patient Perceptions of Integrated Veteran Care (PPIC 2.1) survey instrument (44), which has been validated and psychometrically tested to collect information on patients’ care coordination experiences.

The PPIC 2.1 survey includes items organized under six dimensions of integrated patient care including: (1) *staff knowledge* about the patient’s medical history (3-item dimension), (2) *provider support for the patient*’*s self-directed care* (5-item dimension), (3) *test result communication* (3-item dimension), (4) *provider knowledge* of the patient (5-item dimension), (5) *provider support for medication adherence and home health management* (4-item dimension) and (6) *specialist knowledge* about the patient’s medical history (2-item dimension) (Table 2).

**Table 2:** PPIC 2.1 survey dimensions and sample questions.

Survey dimension	Sample question
Staff knowledge	In the past 6 months, how often did these other staff seem up-to-date about the care you were receiving from this provider?
Provider support for self-directed care	In the past 6 months, did this provider or someone in his or her office ask you about these things that make it hard for you to take care of your health, and did you and this provider or someone in his or her office come up with a plan to help you deal with things that make it hard for you to take care of your health?
Test result communication	In the past 6 months, how often were your test results presented in a way that was easy to understand?
Provider knowledge	How would you rate this provider's knowledge of your values and beliefs that are important to your health care?
Provider support for medication adherence and home health management	In the past 6 months, how often did this provider or someone in his or her office talk with you about what to do if you have a bad reaction to your medicine?
Specialist knowledge	When you see this specialist, does he or she seem to know enough information about your medical history?

See Friedberg et al. (44) for a full list of questions under each survey dimension.

Measures for analysis will be constructed from the six dimensions of the validated questionnaire items of the PPIC 2.1. Additional validated items will verify provider attribution, measure potential differences in individual tendency to give more positive or negative responses using the standardized life orientation test-revised (LOT-R) scale (45), and provide information about Veterans’ sociodemographic characteristics. Surveys will be linked with existing VA and CMS data to verify or ascertain Veterans’ sociodemographic characteristics, and to ascertain their clinical characteristics and healthcare use, including use of care coordination services.

To account for clustering by VA site, we considered an intra-class correlation coefficient of 0.01. Previous work by Benzer et al. (46) using an adaptation of the PPIC instrument on a cohort of Veterans reported mean scores [standard deviation (SD)] in the dimensions of: knowledge integration 3.18 (SD 0.83), support for self-care 3.00 (SD 0.85), and staff knowledge 3.02 (SD 0.91). For our target sample size, we have over 96% power to detect a significant difference between group means as low as 0.3, assuming a maximum SD of 0.9. Power analyses used alpha = 0.05 and were conducted in PASS v16.0.5.62 (47).

#### Analysis

In descriptive analyses, we will compare mean scores for the six survey dimensions between Veterans with and without care coordination services. For individual survey items, we will also compare proportions of the sample of Veterans who reported the most positive rating (top-box score). Next, we plan to estimate six GLMMs, one for each of the six survey dimensions. We will compare mean dimension scores among Veterans with and without care coordination services, adjusting for Veterans’ sociodemographic and clinical characteristics, clustering at the VA facility level, and Veterans’ tendency to give more positive or negative responses using the LOT-R.

### Aim 3: provider perceptions

#### Research question

For Aim 3, our research question is: “What are provider perceptions of care coordination needs assessment tools and process as they relate to concepts of innovation diffusion and care integration?” We expect that providers will highlight the importance of innovation attributes including relative advantage, compatibility, observability, and system innovation fit, and that perceptions about these factors will vary based on provider characteristics and roles.

#### Objective and participants

For Aim 3, we will gauge provider perspectives regarding care coordination tools and processes, and integration with care. We will conduct group and individual interviews with providers based at facilities where adoption of new care coordination processes and assessment tools are underway across the VA (30, 37). As a formative evaluation, these interviews will inform the development and fielding of a provider survey related to determinants of care coordination needs assessment and services, innovation diffusion, and care integration. For group interviews, key informants will be selected based on VA sites’ progress implementing new care coordination tools and processes, and to have a breadth of geographic representation. At each participating site, we will conduct 2–3 group interviews with care coordination team members including lead executives and co-champions; care coordination, care management, or case management leaders (registered nurses and social workers); and care coordinators, care managers, and case managers from different areas and specialties. All group interviews will be conducted with teams as units. Concurrent with group interviews, individual interviews with clinical or administrative leaders less involved in direct care but critical to implementation success will also be conducted, to supplement the positional perspectives included in our sample and will assess respondent characteristics including length of time the respondent has been at their current VA site and their position prior to or concurrent with their care coordination role. We will collect 1–3 individual interviews at 7–10 implementation sites.

#### Measures

Measures for group and individual interviews, which are based on diffusion of innovation theory and adapted from Pankratz et al. (48) perceived attributes of an innovation, will include relative advantage, compatibility, complexity, observability, and trialability (Table 3).

**Table 3:** Perceived attributes of care coordination needs assessment tools and processes, and interview sample questions.

Perceived attribute	Description	Sample question
Relative advantage	Degree to which an innovation is perceived as better than the idea it supersedes.	Are there specific activities that care coordination tools and processes have made harder or easier? What are they?
Compatibility	Degree to which an innovation is perceived as consistent with the existing values, past experiences, and needs of potential adopters.	Tell me about the ways that care coordination processes and tools have changed your workflow. What's different? What's the same?
Complexity	Degree to which an innovation is perceived as relatively difficult to understand and use.	How has the training of staff to use care coordination tools and processes gone? What could have gone better? What more is needed?
Observability	Degree to which the results of an innovation are visible to others.	Have you noticed ways in which the patient experience has changed by your use of care coordination tools and processes? What are they?
Trialability	Degree to which an innovation may be experimented with on a limited basis.	Have you been able to “try out” using care coordination tools and processes alongside old ways you were organizing care coordination, or did you have to switch to a new way of doing things entirely?

Finally, informed by the qualitative interview data, we will develop and field a survey of VA providers. Measures will, again, be drawn from an adaptation of a scale for measuring perceptions of innovation adoption developed by Pankratz et al. (48). Additional questionnaire items will be developed to address attributes related to innovation system fit, organizational power dynamics, and patient centeredness.

#### Analysis

Data from group and individual interviews will be analyzed using a rapid analysis approach and templated notes alongside iterative immersion. Results from this analysis will be used to give formative feedback to operational partners charged with implementing care coordination and needs assessments tools, and to refine the development of the broad provider survey. For the provider survey, we will use descriptive analyses and visualizations to understand survey item responses in relation to provider characteristics and roles, and facility characteristics such as size, patient volume, and type and number of PACT teams for primary and specialty care. Analyses will focus on identifying factors that are most predictive of perceptions of care coordination services and innovation affinity.

## Discussion

This study will evaluate care coordination outcomes and processes in ongoing VA care for Veterans with complex care needs. In a prospective, observational, multiple methods study, we will (1) assess whether receipt of care coordination services impacts healthcare use, costs, and mortality among high-risk, high-need Veterans; (2) examine Veterans’ perspectives about care coordination and integration and whether receipt of care coordination services impacts these perceptions; and (3) explore provider perspectives about care coordination and integration as they relate to elements of innovation diffusion, innovation system fit, organizational power dynamics, and patient centeredness.

The extent to which care coordination is contributing to outcomes among patients with complex care needs is not fully understood, and existing evidence is inconsistent regarding the design and effectiveness of tools to assess care coordination needs (10–12). In addition, the core elements of care coordination have varied considerably across interventions, and lack of standardization of care coordination needs assessment tools and processes has made it challenging to optimize care coordination services delivery based on patients’ needs and preferences (20, 21, 29–32). Our study addresses these gaps by first examining, among Veterans with complex care needs, whether and to what extent receipt of care coordination services impacts their healthcare utilization, health outcomes, and experiences of care coordination and integration. Another novel aspect is that our study assesses the attributes of existing processes used across the VA to assess Veterans’ care coordination needs and preferences. We also explore whether and which attributes are associated with improved care delivery and patient-centeredness. With this, our goal is to inform the design of an implementation study of an improved care model in VA and other health care systems that incorporates patient, provider, and staff perspectives, addresses the identified needs of these groups, and focuses on the care aspects with the greatest potential to improve outcomes.

Strengths of this study include our multidisciplinary research team consisting of clinical practitioners, organizational behavioral experts, implementation scientists, qualitative and quantitative methodologists, and clinical practice partnerships. Members of our research team also have experience working with a wide range of healthcare and related data for research and operations purposes.

In addition, this research will be completed with insight from initiatives underway and led by VA clinical operations offices, in particular the VA Offices of Nursing Services (ONS), Care Management and Social Work (SW), and Integrated Veteran Care (IVC). These clinical operations offices are spearheading efforts to standardize and enhance care coordination practices across the VA, and have collaborated in prior reviews of care coordination research priorities (37, 49). As part of our ongoing work, our team collaborates closely with these operations partners to understand strategies, data resources, and evaluation approaches related to care coordination tools and processes (30). With the ONS and SW, our team participates in their Care Coordination and Integrated Care Management workgroups and Governance Council. With the IVC, we have also worked to understand their needs and weigh in on care coordination needs assessment predictive analytics approaches and risk stratification methods in development. Through these partnerships, we have an opportunity to leverage and gain insight about the ongoing implementation of care coordination initiatives and their relationship with Veteran outcomes. This proactive engagement with operations partners exemplifies the principles of a Learning Health Care System and will further inform approaches to address gaps in care coordination for Veterans (50).

There are limitations to this study design. As an observational study, we will be limited to describing associations rather than causal effects. We also recognize that the CAN score, which is a well-validated tool for identifying one-year hospitalization and mortality risk, is an imperfect tool for assessing patient care needs. Like most health systems, the VA lacks standardized EHR-based assessment tools for care coordination and case management needs. We will include the robust clinical and social information that are available in the VA EHR, and therefore expect to demonstrate the importance of addressing information gaps and in consultation with our operational partners. In addition, although our Veteran survey will assess Veteran's perception of care integration, our study lacks qualitative analysis of Veteran's experiences, which could provide additional insight into whether Veterans are receiving the right care at the right time. Finally, while our study focuses on Veterans healthcare and may have limited generalizability, we may identify elements and processes that can be explored in other settings and populations.

Indeed, our research dissemination process will be broad to motivate and inform further research and quality improvement. We have planned formal information sharing channels with VA stakeholders, including sharing survey results with participants and Veteran Engagement Groups. We will also share progress and early results with our clinical partners and other ongoing research initiatives through VA Health Services Research and Development venues. In addition, we will share our results and insights with the broader healthcare community. While our immediate goal of this research project is to inform development of an enhanced care coordination intervention to improve care integration and Veteran outcomes, we hope that our efforts can build evidence that may inform care coordination planning and implementation in other integrated health systems and community settings.

## Conclusion

By describing our approach and anticipated measures and analyses for this new study, we hope this information will be useful to researchers planning evaluations in other populations facing complex care needs. As we examine the relationship between care coordination processes and health outcomes, assess patient experiences, and gauge provider perspectives, we will share lessons learned. Ultimately, we expect our findings may inform others preparing to implement and evaluate care coordination programs in the VA and in other health systems.
